# Flavonoids as therapeutics for myocardial ischemia-reperfusion injury: a comprehensive review on preclinical studies

**DOI:** 10.1186/s42826-024-00218-2

**Published:** 2024-09-05

**Authors:** Vipin Kumar Verma, Priya Bhardwaj, Vaishali Prajapati, Avantika Bhatia, Sayani Purkait, Dharamvir Singh Arya

**Affiliations:** Department of Pharmacology, All India Institute of Medical Sciences, New Delhi, 110029 India

**Keywords:** Inflammation, Flavonoids, Myocardial ischemia-reperfusion injury, Preclinical studies, Reactive oxygen species, Re-oxygenation

## Abstract

Ischemic heart disease is the most prevalent cause of death worldwide affecting both the gender of all age groups. The high mortality rate is due to damage of myocardial tissue that emanates at the time of myocardial ischemia and re-oxygenation, thus averting reperfusion injury is recognized as a potential way to reduce acute cardiac injury and subsequent mortality. Flavonoids are polyphenol derivatives of plant origin and empirical shreds of evidence substantiate their numerous activities such as antioxidant, anti-inflammatory, anti-apoptotic, and anti-thrombotic activity, leading to their role in cardio protection. Recent investigations have unveiled the capacity of flavonoids to impede pivotal regulatory enzymes, signaling molecules, and transcription factors that orchestrate the mediators participating in the inflammatory cascade. The present comprehensive review, dwells on the preclinical studies on the effectiveness of flavonoids from the year 2007 to 2023, for the prevention and therapeutics for myocardial ischemia-reperfusion injury.

**Graphical Abstract:**

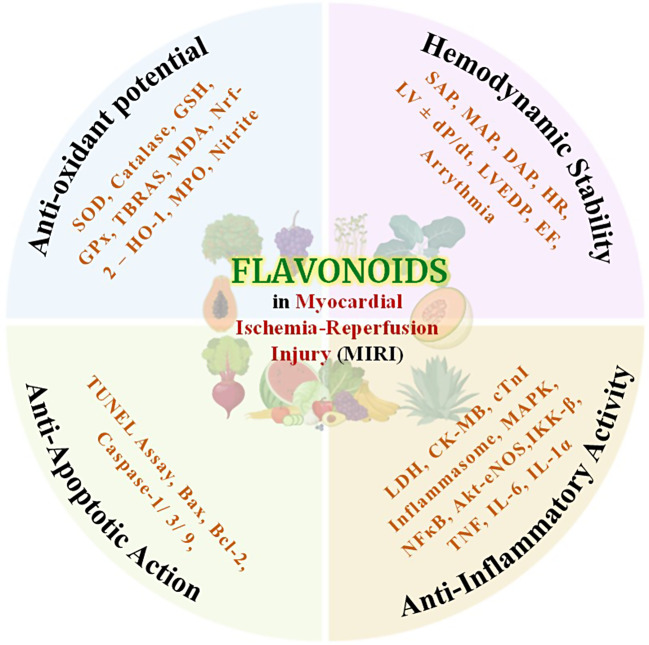

**Supplementary Information:**

The online version contains supplementary material available at 10.1186/s42826-024-00218-2.

## Background

**Fig. 1:**
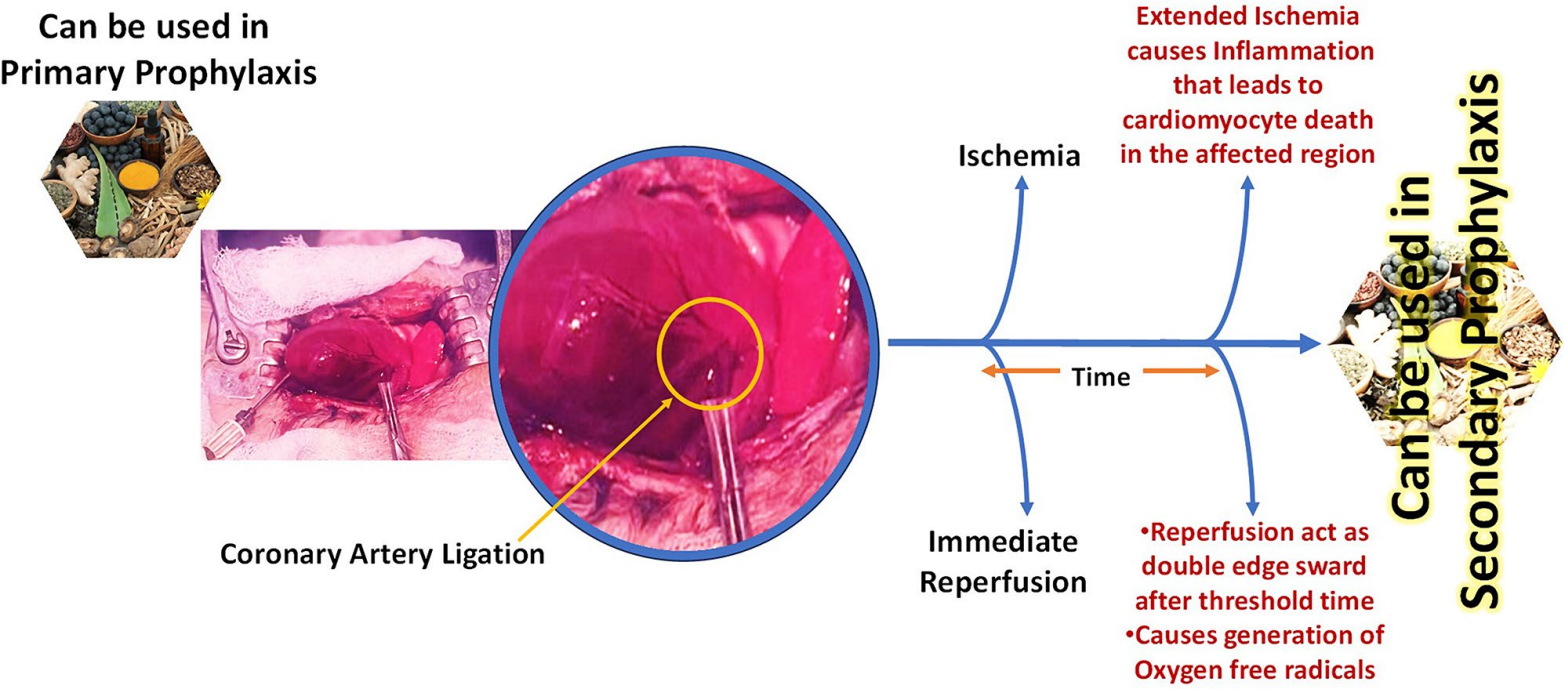
Surgical procedure followed to cause Myocardial Ischemia-Reperfusion Injury (MIRI) in in vivo models

Ischemic heart disease [IHD] is the most prevalent cause of mortality worldwide and accounts for a 2.3-fold rise in the incidence rate of IHD in India [1, 2]. IHD refers to occlusion due to atherosclerosis leading to the inadequate blood supply to the region of the heart or in a broad term, the heart is not getting enough blood and oxygen due to blockage of coronary arteries which transports blood to the myocardium [3]. IHD accounted for 8.9 million deaths in the year 2019, attributed to 16% of total deaths globally [4]. The currently available treatment for IHD is the restoration of blood in the ischemic heart muscles either by surgery or pharmacological therapy [5]. The several available therapy methods that can restore blood flow are coronary artery bypass grafting (CABG), percutaneous coronary intervention (PCI), etc. However, abrupt reperfusion leads to myocardial ischemia/reperfusion injury (MIRI). MIRI causes more structural and dysfunctional damage to cardiomyocytes on resuming blood perfusion than before reperfusion. Also, the rising mortality rate occurs due to myocardial damage that emanates at the time of re-oxygenation of the ischemic myocardium [6]. Therefore, finding a novel therapeutic strategy to prevent patients with a high risk of MIRI is quint essential [7]. Several animal studies and clinical trials have shown that a series of pretreatment methods account for the phenomena of ischemic tolerance. However, among different pretreatment methods such as the pharmacological intervention of beta-blockers, antiplatelets drugs, angiotensin-converting enzyme (ACE) inhibitors, fibrinolytic, calcium channel blockers (CCB), nitrates, cholesterol-lowering agents, exercise, and hypoxia, ischemic pretreatment (IP) has been proved to be the effective protective mechanisms because of its application in the prevention of primary and secondary prophylaxis of IHD [7]. Additionally, the ischemic reperfusion area through surgical procedures or pharmacological treatment causes the oxygen rush in the ischemic area, subsequently leading to oxidative stress by the formation of oxygen free radicals/ROS. Therefore, averting reperfusion damage is a pivotal way to overcome morbidity of acute cardiac injury as discussed in Fig. 1 [8].

Flavonoids have the inherent capability to combat numerous human diseases [9]. On a global scale, the rising prevalence of overweight and obese individuals has led to a significant surge in concurrent medical conditions, underscoring the imperative for improved therapeutic approaches. The positive influence of flavonoids on obesity and associated ailments is attributable to their anti-inflammatory action [10]. Inflammation-evoked reactions/responses significantly participate in the pathogenesis of several ailments such as diabetes, asthma, cardiovascular disorders, and cancer. The inflammatory cascade is a complex interaction involving the recruitment of various immune cells, driven by pro-inflammatory triggers. These immune cells subsequently generate chemokines and pro-inflammatory cytokines that serve as chemo-attractants for lymphocytes, thereby activating adaptive immune response. Within the context of this inflammatory cascade, the generation of oxygen free radicals, reactive nitrogen species (RNS), and a diverse array of proteases ensues, each of which holds the potential to precipitate tissue damage, fibrogenesis, and cellular proliferation, broadly can contribute to the perpetuation of chronic inflammation [11].

**Fig. 2:**
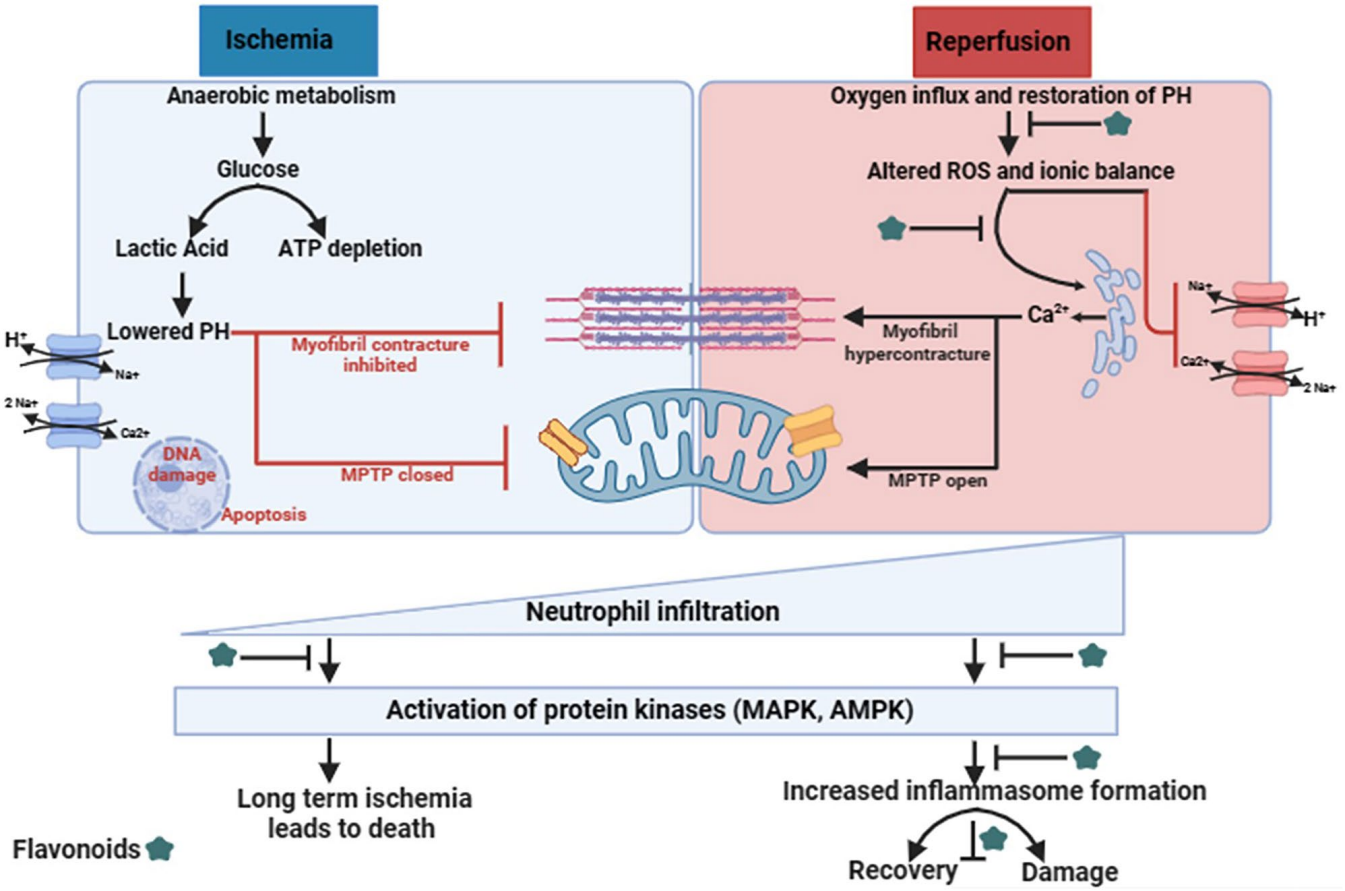
Mechanism involved in the damage from Myocardial Ischemia-reperfusion injury and the role of flavonoids at various point in different studies

As inflammation already initiates during ischemic events, the subsequent reinstatement of blood circulation and oxygen supply amplifies the activation of inflammatory signaling pathways. Ongoing research endeavours are dedicated to probing the inflammatory molecules and cascade involved in ischemic injury, with a particular focus on pivotal factors such as interleukins (IL), neutrophils, and inflammasomes (Fig. 2) [12]. Also, it has been demonstrated that consumption of flavonoids protects against incidences of IHD, suggesting that flavonoids may enhance tolerance to MIRI [8, 13]. The present review will provide insight into the preclinical studies towards the effectiveness of flavonoids in IR injury. These flavonoids have an effective role in cardioprotection and could be taken further to the clinics after well-designed clinical studies.

## Main text

Myocardial ischemia is characterized by damaged myocardial tissue. ROS causing severe myocardial cell damage has been demonstrated in the chick model of simulated IR injury in cardiomyocytes [14]. The reperfusion in the ischemic region of the heart paradoxically initiates myocardial injury characterized by myocardial apoptosis/ necrosis/ necroptosis and pyroptosis/ ferroptosis. Broadly, during reperfusion of ischemic myocardium, oxidative stress, and ionic disturbance are primarily attributed to myocardial IR injury. During reperfusion, ionic disturbances and increased oxygen free radicals activate signaling pathways leading to cardiomyocyte death in severe cases [15]. This cell death releases damage-associated molecular patterns (DAMPs), mitochondrial DNA fragmentation, high mobility group box 1 protein (HMGB1), ATP, and calcium. These DAMPS activate TLR9 and NLRP3-inflammasome formation, triggering inflammatory responses. Subsequently, nuclear factor-κB (NF-κB) and myeloid differentiation primary response gene 88 (MyD88) pathways get activated resulting in the release of inflammatory molecules like interleukin-1β (IL-1β), monocyte-chemoattractant protein 1 (MCP1), tumor necrosis factor (TNF), IL-6, and IL-18. Furthermore, activation of inflammasome augments secretion of IL-1β and IL-18 via cardiac fibroblasts, leading to apoptosis of cardiomyocytes by increased expression of caspase-1 known as pyroptosis. In addition, leukotriene B4 (LTB4), cytokine-induced neutrophil chemoattractant 1 (CINC-1), macrophage inflammatory protein-2α (MIP-2α), complement 5a, IL-8 and CXCL8 amplifies recruitment of neutrophils to infiltrate in damaged area after the onset of ischemia which further leads to overproduction of ROS and releases granular components composed of proteases and myeloperoxidase, to remove apoptotic bodies as well as necrotic debris.

Despite neutrophils, activated complement constituting 30 proteins and protein fragments also get infiltrated at the reperfused area resulting in augmentation of inflammation and damage, derived by complement pathway [Fig. 2]. Further, monocyte recruitment occurs at the site of the reperfused area due to chemokines (MCP1) and complement fragments (C3a, C4a, and C5a). Importantly, monocytes arise from the bone marrow and are secreted in the bloodstream via 2 ways: (a) Ly6C^hi^ monocytes are characterized by inflammatory activity, released in blood stream and peak after 3–4 days of post-myocardial infarction. (b) Ly6C^low^ monocytes are characterized by anti-inflammatory activity and peak on the 7th day after myocardial infarction. The Ly6C^hi^ monocytes acts by removing debris through phagocytosis at the reperfused damaged area. In addition, monocytes (Ly6C^hi^) differentiate into M1-type macrophages, characterized by phagocytic activity, and produce ROS, resulting in enhanced inflammation. Later, Ly6C^low^ monocytes start infiltrating in the reperfused damaged region and M1-type macrophages differentiate into M2-type macrophages resulting in suppression of T-cell activation by secreting TGF-β and IL-10. In addition, TGF-β functions in tissue remodelling and vascularization. Moreover, Th1-inducing factors prevent a shift of M1 to an M2-type of macrophages thus reducing the healing potential of chronic myocardial. Thereby, IR injury emanates into two phases: acute and delayed phase. During the acute phase, oxidative stress is primarily generated through the mitochondrial electron transport chain (ETC) and xanthine oxidase pathway. Inflammatory reactions occur due to cytokines from damaged cells leading to enhanced ROS levels, later during the delayed phase [16]. At each phase during the pathophysiology of IHD, flavonoids could be used for the amelioration of ischemic reperfused tissue.

Flavonoids are polyphenolic compounds naturally found in plant sources including vegetables and fruits. Several preclinical studies have evidenced the antioxidant activity of these compounds by in vitro and in vivo models of oxidative stress. Also, clinical studies have demonstrated the consumption of flavonoids from fruits, vegetables, and tea at recommended doses decreases the incidence of IHD [17, 18].

### Classifications of flavonoids

Over 4000 different flavonoid compounds have been identified from plants. These flavonoid compounds based on their chemical structure are categorised into flavonols, flavones, isoflavones, flavanones, and flavanonols as given in Table 1. Phenol benzopyrone skeleton (C6-C3-C6) remains the common entity between these groups.

**Table 1:** Different Chemical Class of flavonoids, constituents and its sources

Chemical Class	Constituents	Common Plant Source
Flavanols	Catechin, Gallo catechin	Tea, Apple
Flavanol	Quercetin, Myricetin, Kaempherol, Rutin	Tea, Apple, Red wine, Tomato, Onion and Cherry
Flavones	Apigenin, Chrysin, Luteolin	Parsley and Thyme
Isoflavones	Genistein, Formononetin, Daidzein, Glycitein	Soya bean and other Legumes
Flavanones	Hesperidin, Narigenin	Oranges and Grapefruit
Flavanonols	Taxifolin	Lemon and Sour orange

### Mechanisms associated with flavonoids in the prevention of IR injury

#### Free radicle scavenging and antioxidant activity

Previous studies have reported that flavonoids exhibit ROS-scavenging properties, and reduce oxidative damage during myocardial IR injury. Flavonoids also scavenge peroxy-nitrite, superoxide, and peroxide radicals. Despite this, flavonoids prevent the Fenton reaction by forming complexes with iron [19]. Fanton reaction is an advanced oxidation process (AOP) that decomposes hydrogen peroxide using iron and generates hydroxyl ions [20]. Xanthine oxidase and NADPH oxidase play vital role in the generation of oxygen free radical. Many flavonoids such as apigenin, luteolin, quercetin, kaempferol, and myricetin, have been demonstrated to impede these oxidases and subsequently inhibit the production of ROS [21].

#### Chelation of transition metals

Flavonoids have been shown to chelate iron and copper which plays an important role in free radical generation. Chelation of iron leads to the prevention of free radical generation by the Fenton reaction [22].

#### Effect on myocardial apoptosis

Several preclinical studies have demonstrated that flavonoids have a role in cardio protection by depleting pro-apoptotic factors (BAX, BAD, and BID), and cytosolic proteases including caspase-3, caspase-8 and caspase-9. Moreover, flavonoids like fisetin, kaempferol, mangiferin, hesperidin, naringenin, baicalein, genistein, luteolin, morin, nobiletin, quercetin, etc. act by inhibiting cytoplasmic proteases.

#### Anti-inflammatory activity

**Fig. 3:**
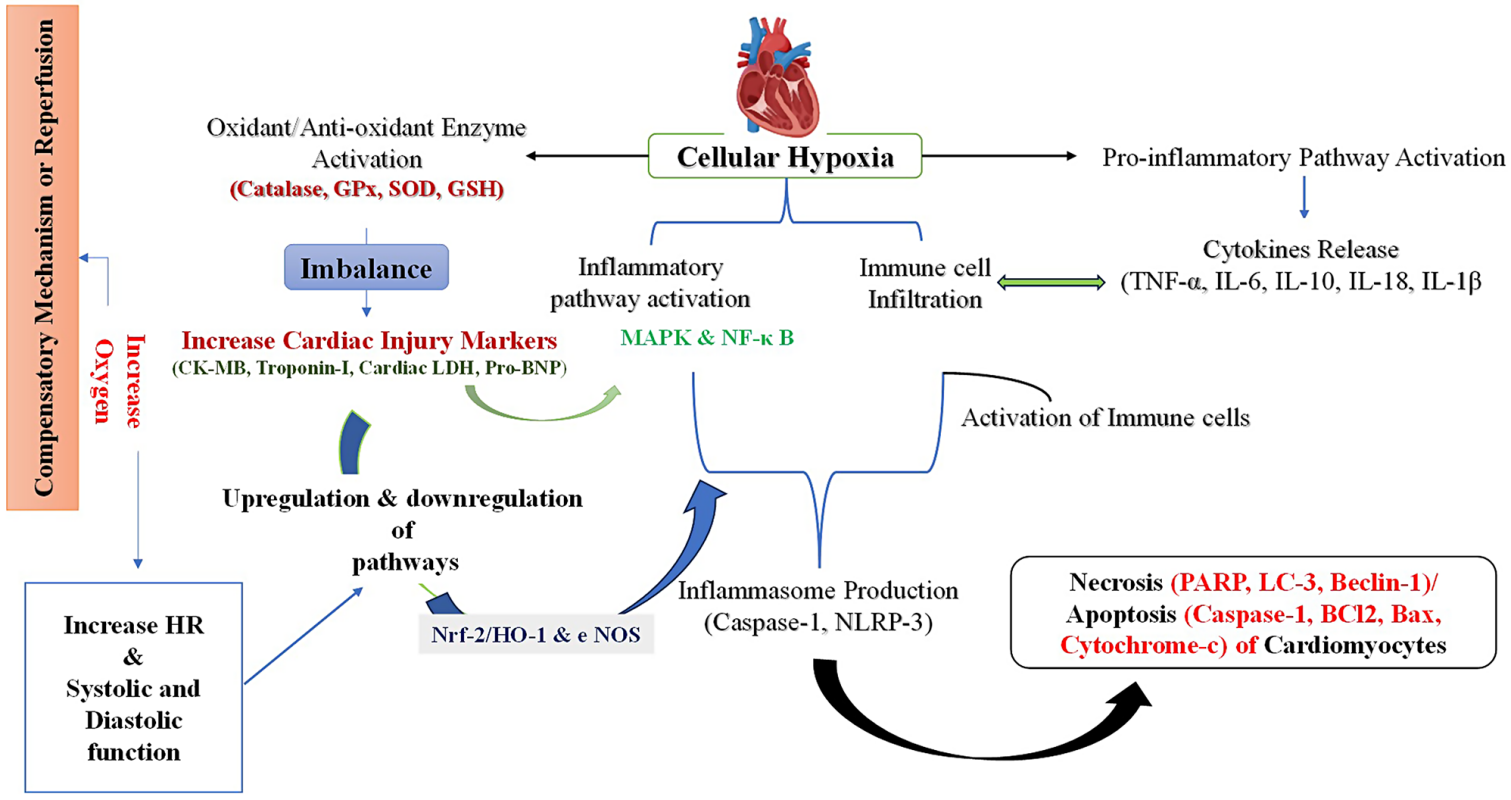
Key Molecular pathways involved and studied to investigate the effect of flavonoid in myocardial ischemia-reperfusion injury (MIRI) in vitro, in vivo, and ex vivo models of myocardial Infarction

Several flavonoids possess anti-inflammatory and anti-aggregatory properties. Studies revealed that the flavonoids inhibit matrix metalloproteinases (MMPs), which participate in tissue remodelling by degrading extracellular matrix components. The increased plasma levels of MMPs have been reported during myocardial IR injury [23]. Flavonoids such as fisetin, kaempferol, baicalein, diadzein, genistein, luteolin, morin, and quercetin work by suppressing the activation of NF-κB leading to inhibition of pro-inflammatory cytokines (IL-6 and TNF-α). Furthermore, myocardial IR injury leads to acute inflammation in the myocardium where neutrophils infiltrate and subsequently progress the myocardium injury. Flavonoids have been demonstrated to protect against myocardial IR injury by inhibiting pro-inflammatory cytokines (IL-6, IFN-γ, and TNF-α). Several evidences have shown that flavonoids act as an anti-inflammatory via inhibiting activation of the NF-κB and AP-1 transcription factors [23]. The targeted molecular pathway of flavonoids is explained in Fig. 3.

### Flavonoids and molecular pathways associated with the prevention and therapeutics of myocardial IR injury

**Fisetin** (3,3,4,7-Tetrahydroxyflavone) is a flavone isolated from vegetables and fruits. An ex vivo study on an isolated rat heart showed that a fisetin dose of 20 mg/kg by intraperitoneal route significantly decreases myocardial IR injury by its antioxidant activity and downregulating glycogen synthase kinase 3 beta (GSK-3B) [24]. Furthermore, an in vitro study conducted on H9c2 cardiocytes reported that fisetin treatment at a concentration of 15 µM stimulates the viability of cardiomyocytes, inhibits apoptosis, and activates cytosolic caspases (caspase 3, 8, and 9), reduces the generation of ROS and protects from DNA damage [25]. An in vivo study demonstrated that fisetin at a dose of 10 mg/kg and 20 mg/kg protects against myocardial IR injury by downregulating RAGE and NF-κB levels [26].

**Kaempferol** (3,5,7-Trihydroxy-2-(4-hydroxyphenyl)-4 H-1-benzopyran-4-one) is a flavanol and isolated from various plants such as Witch-hazel, Delphinium, and grapefruit [27]. Several preclinical studies have demonstrated that kaempferol treatment significantly protects against myocardial IR injury via reducing apoptosis, GSK-3 beta activity and inhibiting the expressions of endoplasmic reticulum (ER) stress proteins [28–30]. Numerous in vitro studies reported that kaempferol treatment attenuates myocardial IR injury by reducing pro-inflammatory cytokines (IL-1β, IL-6, and TNF-α), and by inhibiting pro-apoptotic proteins (Bax & caspase-3) and stimulating expression of anti-apoptotic protein Bcl-2 [31, 32].

**Mangiferin** (1,3,6,7-Tetrahydroxyxanthone-C2-β-D-glucoside) C-glucosyl xanthone) is found in leaves, stem bark, fruit peels, and roots of *Mangifera indica* (mango) with antioxidant, and antidiabetic activity [33]. Numerous studies have revealed that treatment with mangiferin protects from IR injury by reducing the phosphorylation of p38 and JNK and increasing the phosphorylation of ERK 1/2. Mangiferin treatment also reduces and increases the expression of pro-apoptotic and anti-apoptotic proteins respectively. [34–36].

**Hesperidin** (30, 5, 9-Dihydroxy-40-methoxy-7-Orutinosyl) is a flavanone extracted from citrus fruits, and has anti-inflammatory, antioxidant, and anticancer properties. Plethora of preclinical data reported that hesperidin improves myocardial IR injury by decreasing the plasma levels of oxidative stress and pro-inflammatory cytokines [37–40]. Other preclinical studies reported that hesperidin play a role in cardioprotection by inhibiting HMGB1 and activating PI3K/AKT pathways [41, 42].

**Naringenin** (4,5,7-Trihydroxy flavanone) is a flavanone found in citrus fruits, and characterized by antioxidant, anti-inflammatory, anti-apoptosis, and anticancer properties. Several in vitro and ex vivo studies revealed that naringenin attenuates myocardial IR injury by inhibiting mitochondrial oxidative stress and endoplasmic reticulum (ER) stress [43–46].

**Catechin (**flavan-3-ol**)** is a bioactive polyphenol found in green tea and characterized by antioxidant, antioncogenic, and antiviral properties. A study reported that baicalin protects against myocardial IR injury when given just after reperfusion [47]. Another in vitro by Cong and his co-workers showed that treatment with catechin augments mitochondrial function and reduces apoptosis by encouraging activation of Akt / Gsk-3β [48]. Recently, a meta-analysis study demonstrated that epigallocatechin gallate (EGCG) significantly alleviates oxidative stress, myocardial injury enzyme, and cardiac function in myocardial IR injury animal models [49].

**Daidzein** (7,4′-Dihydroxyisoflavone) is a phenolic compound that belongs to the phytoestrogens class and is found in soybeans & soy products and plants such as the Thai Kwao Krua [50]. A preclinical study conducted on an animal model of IR by Kim et al., in 2009 reported that daidzein depletes the plasma levels of TNF-α, IL-6, myeloperoxidase, catalase activity along with reduced malondialdehyde levels. Also, it inhibits myocardial apoptosis via reducing DNA strand breaks, and caspase-3 activity, along with downregulation of activated NF-κB transcription factor [51]. Moreover, it has been demonstrated to attenuate doxorubicin-induced cardiac injury via impeding apoptosis and autophagy [52]. A previous study by Shu et al. reported that daidzein decreases the activation of TGF-β1-induced cardiac fibroblast by TGF-β1/ SMAD2/3 signaling pathways [53].

**Genistein** (4′,5,7-Trihydroxyisoflavone) is a polyphenolic isoflavone and is extracted from dietary vegetables, such as fava beans and soybeans. Several preclinical studies have reported that genistein attenuate myocardial IR injury by decreasing myocardial apoptosis (lower Bcl2/ Bax ratio and Bax expression) and necrosis. Apart from this, genistein also reduces the pro-inflammatory cytokines such as IL-6, IL-8, IL-10, and TNF-𝛼 as evidenced from the previous studies [54, 55].

**Luteolin** (3′,4′,5,7-Tetrahydroxyflavone) is a flavone, isolated from leaves and rinds, ragweed pollen, broccoli, pepper, thyme, celery, and barks [56]. Primitive studies reported that luteolin ameliorates myocardial IR injury through reduced myocardial necrosis and apoptosis [57]. It has been shown that luteolin acts by upregulating and downregulating the expression of anti-apoptotic protein (Bcl-2) and pro-apoptotic protein (BAX) respectively. Preclinical studies showed that the anti-apoptotic and anti-inflammatory properties of luteolin play a vital role in the improvement of myocardial IR injury [58, 59]. Furthermore, previous studies have demonstrated the inhibitory effect of luteolin on IR injury-induced SERCA2a activity [57, 60, 61].

**Morin** (2′,3,4′,5,7-Pentahydroxyflavone) is a natural polyphenol and is extracted from stems, branches, leaves, and fruits of different plants. An in vitro study demonstrated that morin ameliorates myocardial IR injury via its anti-apoptotic activity and by impeding the opening of myocardial mitochondrial permeability transition pores (MPTP) [62]. Morin functions via decreasing cytosolic caspase-3 & Bax and augmenting the anti-apoptotic protein levels (Bcl-2). Moreover, it also reduces myocardial inflammation by regulating inflammatory mediators such as TNF-α, IKKβ, NFκB, and IL-6) in the myocardium [62–64].

**Nobiletin** (O-methylated flavone) is a flavonoid found in citrus peels. An in vitro study reported that nobiletin improves myocardial IR injury by downregulating pro-inflammatory cytokines levels involving TNF-α, IL-6, IL-1β, and MDA levels [65]. In addition, nobiletin reduces the Bcl-2 level while increasing the Bax and caspase − 3 levels. Effects of nobiletin in cardiomyocytes were shown to be accomplished by stimulating the Akt/GSK-3β pathway. A preclinical study reported that nobiletin improves myocardial IR injury by upregulating p-PI3K & p-AKT levels [66, 67].

**Quercetin** (3,5,7,3’,4’-Pentahydroxyflavone) is a polyphenolic compound found in onions, berries, grapes, broccoli, cherries, and citrus fruits and comprises different biological activities including antioxidant, anticoagulant, and anti-inflammatory activities [68, 69]. Various studies have demonstrated the role of quercetin in improving myocardial IR injury by stimulating the PI3K/Akt signaling pathway, and peroxisome proliferator–activated receptor gamma (PPAR-γ). Also, evidence from in vitro study have proved that quercetin improved myocardial IR injury by reducing the pro-inflammatory cytokines (IL-10 and TNF-α) [70]. A study showed quercetin in combination with cinnamaldehyde improves inflammation, myocardial infarction, and apoptosis in isoproterenol-induced rats via cleaved caspase-3 signaling, NF-κB, and P65 molecules [71]. Various preclinical studies evaluated the potent role of flavonoids in the prevention and therapeutics of IR injury along with doses used and results obtained are summarized separately for in vivo (Table 2), ex vivo (Table 3), and in vitro (Table 4).

**Table 2:** Preclinical studies evaluating the potential of flavonoids in the prevention and treatment of myocardial ischemia-reperfusion injury (MIRI) *in-vivo *

Flavonoids	Dosing Pattern	Animal	MIRI protocol (mins)	Inference	References
**Fisetin**	20 mg/kg/day orally for 28 days	Wistar Rats	45–60	• ↑ SOD, Catalase, GSH • ↓ TBARS, LDH, CKMB • Downregulation of RAGE and NF-κB	[26]
**Kaempferol**	20 mg/kg/ day; i.p. for 15 days	Wistar Rats	45–60	• ↓ CKMB & LDH, TNF-𝛼, IL-6 & NF𝜅B • Inhibition of active JNK & p38 proteins • activation of ERK1/2, pro-survival kinase • ↓ expression of pro-apoptotic proteins (Bax and Caspase- 3)	[31]
	20 mg/kg/day; i.p. for 28 days	Diabetic Wistar Rats	45–60	• ↓ CKMB, LDH, TNF-α, IL-6, NF-κB • ↓ Bax & Caspase-3 • ↓ AGE-RAGE/ MAPK induced oxidative stress & inflammation • ↑ Level of anti-apoptotic protein (Bcl-2)	[32]
**Mangiferin**	40 mg/kg/ day, i.p. for 15 days	Wistar Rats	45–60	• ↓CKMB, LDH, TNF-𝛼, IL-6, TGF beta level • Normalizing oxidative stress • ↓ Phosphorylation of p38, & JNK • ↓ Bax & Caspase-3, but ↑ Bcl-2 expressions • ↑ phosphorylation of ERK1/2	[34]
	40 mg/kg/day; i.p. for 28 days.	Diabetic Wistar Rats	45–60	• ↓ CKMB, LDH, and oxidative stress • ↓ TNF-𝛼, IL-6, Bax & Caspase-3 • ↑ Bcl-2 & ERK1/2 levels • Inhibited AGE-RAGE, JNK, and p38 activation	[35]
	50 mg/kg/ day orally for 4 weeks	SD Rats	30–120	• ↓ CK-MB, LDH, MDA, and ↑ SOD • ↓ Bax, caspase-3, Caspase-9 and ↑ Bcl-2 level. • Up-regulate Nrf-2 and HO-1	[65]
**Hesperidin**	100 mg/kg/ day orally for 15 days	SD Rats	30–60	• ↑Tissue nitrite, GSH, Catalase & SOD levels • ↓ MDA, TNF, CK-MB, & MPO activity • ↓ Arrhythmias and apoptosis	[37]
	100 mg/kg/ day orally for 14 days	Wistar Rats	45–60	• ↓ Oxidative stress markers and TNF-𝛼, IL-6 • ↓ Expression of Bax and ↑ Bcl-2 • ↓ Levels of CKMB and LDH	[38]
	200 mg/kg/ day orally for 3 days	SD Rats	30–240	• ↓ Infarct size, LDH, CKMB, TNF-𝛼, and IL-6 • Inhibited apoptosis (↓ Bax and ↑ Bcl-2), inflammation & oxidative stress • ↓ HMGB1 & ↑ p-Akt expression	[41]
	200 mg/kg/ day orally for 3 days	SD Rats	30–240	• ↓ Myocardial infarct size, myocardial damage • ↓ Serum CK-MB & cTnI. • Activation of the PI3K/Akt/mTOR pathway • Inhibits excessive autophagy.	[42]
**Naringenin**	50 mg/kg/ day orally for 5 days	SD Rats	30–240	• Improve hemodynamic • Attenuates myocardial apoptosis & infarction. • ↓ Superoxide generation, MDA level, • ↓ gp91phox, p-ERK, IRE1α, EIF2α, ATF6, & CHOP • Activated myocardial cGMP-PKGIα signalling	[45]
**Catechin**	250 mg/kg/ day intragastric for 10 days	SD Rats	30-1440	• Improved Heart function, ↑ EF, ↓ infarct size • Inhibit necrosis and infiltration of inflammatory cells in the myocardium	[48]
**Daidzein**	5, 10 mg/kg, *i.p.* 1 h Pre-op	SD Rats	25–60/120	• ↓ MDA, MPO, TNF, IL-6, & neutrophil infiltration • Inhibit myocardial apoptosis cleaved caspase-3 • Inhibition of NF-kB activation	[51]
**Genistein**	0.25, 0.5, 1.0, 1.5, 3, and 5 mg kg) *i.v.*, 5 min after ischemia	SD Rats	45–300	• Improve Hemodynamic function • ↓ Myocardial necrosis, MPO, CPK, TNF, and blunted ICAM-1 expression • Reduced TNF in intraperitoneal macrophages	[72]
	1.0 mg/kg/ day *i.v.* 5 min Pre-op	Rabbit	45–180	• Preserve hemodynamic • ↓ Infarct size, apoptosis • ↓ Fas and Bax expression; ↓ Bcl-2/Bax ratio	[73]
	20, 40, and 60 mg/kg/ day orally, 5 days Pre-op	SD Rats	30–60	• ↓ Infarct size, preserve Histopathology and hemodynamic • ↓ CK, LDH, GSH, MDA and ↑ Catalase, SOD • ↓ IL-6, IL-8, IL-10, TNF-𝛼 and Suppress P2 × 7/NF-𝜅B	[74]
**Luteolin**	0.01, 0.1, 1.0, and 10.0 µg/kg *i.v.*15 mins Pre-op	SD Rats	30–30	• ↓ Arrhythmia duration after Ischemia and reperfusion dose-dependent • ↓ LDH, MDA & NO levels. Luteolin (@ 10 µg/kg) • Down regulated inducible NO synthase protein & mRNA expression in occluded zone	[75]
	10 µg/kg i.v. (tail) for 3 days Pre-op after 8 weeks of diabetic induction	SD Rats	30–180	• ↓ Arrhythmia incidence & preserve LV function • ↓ LDH, MPO and infarct size • ↓ Cleaved caspase-3, ↓ Bax/ Bcl-2 ratio & ↑ FGFR2 & LIF expression • ↑ p-Akt & p-BAD, ↓ IL-6, IL-1α & TNF-α levels	[76]
	Pretreatment @ 200 mg/kg orally for 2 weeks	SD Rats	• 30 − 0/30/60/ 120/360/ 720/1440/4320/7200/10,080 • 60 − 30	• ↓ Infarct size, LDH release • ↑ Bcl-2, ↓ Cleaved caspase-3 and Bax. • Improved SERCA2a activity • ↑ p-Akt (308)/ Akt and p-Akt (473)/ Akt protein expression	[61]
	40/ 80/ 160 mg/kg), orally for 7 days	SD Rats	30-1440	• Preserve hemodynamic dose-dependently • ↓ AST, CK-MB, LDH, IL-1β, IL-18 & TNF-α • ↓ TLR4, MyD88, p-IKKα/ IKKα, p-IKKβ/ IKKβ and p-NF-kB/ NF-kB • ↓ NLRP3, ASC and Caspase-1	[77]
	Pretreatment @ 5, 10, 15, 20, 25 µg/kg, *i.v.* (tail) for 3 days	C57BL/6	30-1440	• Upregulate JPX to improve cardiac function • Inhibit apoptosis: ↓ Bax, Caspase-3 & ↑ Bcl-2 • Upregulate SERCA2a and SUMO1 • ↓ Myocardial infarct size • Improve hemodynamic,	[78]
	5,10, 20 mg/kg/day *i.p.*, 15 min Pre-op	SD Rats	30-1440	• Maintains hemodynamic • ↓ Infarct size, CK-MB, LDH, AST, ROS, MDA • ↑ GSH, SOD • Inhibit apoptosis in a dose-dependent manner	[79]
**Morin**	Pretreatment @ 40, and 80 mg/kg	Wistar Rats	45–60	• ↓ CKMB & LDH. • ↓ Bax, caspase-3 and TUNEL positive cells; while ↑ Bcl-2 • Inhibit MAPK inflammation pathway • ↓ TNF-α, IL-6, NFκB, IKKβ	[64]
**Nobiletin**	15, 30, and 45 mg/kg intravenously (Tail) at the beginning of the reperfusion.	SD Rats	30–120	• ↓ Disease score, CK-MB, and LDH, improve histopathology • ↓ Number of apoptotic cells and apoptotic index • Improve Ejection fraction and fractional shortening • Relatively decreases mRNA/protein expression of myocardial GRP78, CHOP & caspase-12 • Pre-treatment activates Akt/PI3K pathways.	[66]
	7.5, 15, and 30 mg/kg/ day i.p for 21 days	SD Rats	30–120	• Improve hemodynamic functions • Alleviates myocardial Infarction and Fibrosis • Inhibit MAPK-induced inflammation	[39]
**Quercetin**	1 mg/kg 5 min Pre-op	Rabbits	30–720	• ↓ NOX2, eNOS, iNOS mRNA & protein expression	[80]
	Pretreatment @ 1 mg/kg/ day, *i.v.*	SD Rats	30–720	• Improve hemodynamic (↓ LVEDP; ± dp/dt max) • Attenuate plasma levels, and protein as well as RNA expressions of TNF, IL-10	[70]
	10 mg/kg 5 min before reperfusion	SD Rats	30–120	• Reduced infarct size with ↓ CKMB & LDH • ↓ Apoptosis: ↓ caspase- 3 activations, ↓ Bax, ↑ Bcl-2, ↑ Bcl-2/Bax ratio • Activate PI3K/ Akt signaling pathway	[81]
	Pretreatment @ 250 mg/kg/day for 10 days	Wistar Rat	30–240	• Preserve hemodynamic (LVSP, LVEDP, ± dp/dt max) • ↓ Apoptosis rate, CK, AST, LDH, and MDA. • ↑ GSH, SOD, CAT, GSH-Px, and GR activity. • ↓ TNF, CRP, IL-1β, apoptotic cells, ↓ cleaved Bax and ↑ Bcl-2, as well as p-Akt	[82]
	Pretreatment @ 250 mg/kg for 10 days	C57/BL6 mice	30-1440	• ↑ PPARγ, ↓ infarct size, ↑ EF & FS • ↓ AST, CK-MB, cTnT, LDH, MDA, SOD, GPx • ↓ iNOS, cleaved caspase 3 expression • ↓ TUNEL positive cells,	[83]
	25, 50, and 100 mg/kg orally for 7 days	SD Rats	30–120	• Improve pathological myocardial architecture. • ↓ MDA, LDH • ↓ Cell apoptosis rate, Bax expression • ↑ SIRT1, PGC-1a, Bcl‐2	[84]

*Note* MIRI protocol section showed the time duration in minutes (mins) for ischemia followed by reperfusion (e.g. 45 min ischemia – 60 min reperfusion)

**Table 3:** Role of selected flavonoids in the prevention/ treatment of MIRI in ex-vivo experiments (Langendroff-model)

	Dosing Pattern	Study Protocol	Inference	References
**Luteolin**	Pretreatment @ 40 µmol/l for 30 min	(Wistar rats) (MIRI 30–120)	• Improved LVF, HR, LV dp/dt, LVEDP • ↓ Infarct size, LDH activity, • ↓ Apoptosis (lower Bax more Bcl-2), ↑ Bcl-2/Bax ratio. • ↓ Phosphorylation of P38, JNK, but ↑ ERK • ↓ p-PP1a while ↑ p-PLB and SERCA2a levels	[60]
	100 mg/kg/day, i.p. for 2 weeks	Hypercholesterole-mic rat (MIRI 30–120)	• Improve LVF and cardiac tissue viability, • ↓ LDH release & MDA level • ↑ p-Akt & p-GSK3β expressions & activate Nrf2 • ↑Akt-mediated Nrf2 antioxidant & inhibit mPTP	[85]
	100 mg/kg/day, intragastric for 2 weeks	SD Rats (6 week diabetic) (MIRI 30–120)	• Improve LVF, ↓ LDH, MDA, 8-OHdG • ↑ SOD, GPx, Catalase, and HO-1 • ↑ Nrf-2/Histone H3, ARE-Luciferase activity • Enhancing eNOS-mediated S-nitrosylation of Keap1	[86]
**Morin**	10, 20, and 40 mg/kg i.p. OD for 5 days before surgery	SPF Wistar Rats (MIRI 30–60)	• Improve coronary circulation • ↓ Infarct size and improve MPTP • ↑ Bcl-2, while ↓ Bax and Bax/Bcl-2 mRNA expression and apoptosis rate • ↓ Cytochrome c, APAF-1, Cleaved caspase 9/ 3 levels	[62]
**Naringenin**	100 mg/kg i.p.; 2 h before heart excised	Wistar Rats	• Improve left ventricle function, • Activate mitoBK K-channels for cardioprotection	[87]
	1.25, 2.5, 5, 10, 20, 40 µmol/L; 5 min before ischemia	SD Rats (MIRI 30–60)	• > 2.5 µmol/L improved left ventricular function • ↓ LDH in coronary effluent • ↑ SOD, ↓ MDA, and reduced myocardial infarct area. • Activate ATP-sensitive potassium channels in both cell and mitochondrial membrane,	[43]
**Quercetin**	0.033 mg/ kg/day 4 days	SD Rats (MIRI 22–30)	• Improve hemodynamics throughout ischemia and reperfusion improved mitochondrial function after I-R.	[88]
	50 mg/ for 7 days and 15 mmol/L 30 min before ischemia	SD Rats (MIRI 60–60)	• Stabilize hemodynamic • ↓ LDH, CK-MB and cTnI levels from chronic pretreated groups were significantly lower than acute group • ↓ MDA, ↑ GSH, GR	[89]
	20 mg/kg/day for 4 weeks	Wistar rats *Juvenile & adult* (MIRI 25–40)	• Improved post-ischemic recovery of ± dP/dt max, LVDP, in juveniles difference was insignificant in adults	[90]
	Treatment @ 50 mg/kg for 5 days after surgical occlusion of coronary artery	SD Rats (MIRI 30–30)	• Reduced infarct size, and attenuated coronary flow and myocardial contractability • ↓ TNF-α, IL-6, IL-1β, LDH and CK • ↓ Activation of HMGB1/ TLR/ NFκB pathway in LAD ligated heart and global ischemia in isolated heart ↓ TNF-α, IL-6, IL-1β, in cultures cell supernatant	[69]
	100 nM for 10 min before reperfusion	Wistar Rats (MIRI 30–55/45)	• Significantly improve LVF ↓ CK, TNF, IL-6, IL-1β,	[91]

*Note* MIRI protocol section showed the time duration in minutes (mins) for ischemia followed by reperfusion (e.g. 45 min ischemia – 60 min reperfusion)

**Table 4:** In-vitro studies evaluating the potential of flavonoids in the prevention and treatment of myocardial ischemia-reperfusion injury (MIRI) mimicking hypoxia-reoxygenation (H/R)

Flavonoid	Dosing & H/*R* protocol	Cell line	Inference	References
**Catechin**	25µM; 72 h before H/R (60–180)	Chick embryo Cardio- myocyte	• High free radical scavenging activity • ↑ Cell viability • Prevents MIRI damage	[47]
	1, 5, 10, 20, 50 µmol/L; 30 min before H/R	H9c2	• ↑ Cell viability • ↑ CREB & ↓ down-regulated lncRNA MIAT expression • Improve mitochondrial function & relieved apoptosis through promoting Akt/Gsk-3β activation	[48]
**Daidzein**	2, 5, 10, 20, 50, 100 µM	HUVECs	• Showed maximum cell viability & Cell survival @ 5 µM • Inhibit NF-κB luciferase activity	[51]
**Luteolin**	0.5, 1.5, 2.5, & 5.0 µg/ml; 24 h before H/R (180 − 120)	Adult SD rat cardio-myocytes	• ↓ LDH levels & Improve hemodynamic • Less myocardial shortening • ↓ Bax & caspase-3; ↑ Bcl-2; Bax/ Bcl-2 ratio)	[92]
	5, 10, 20 µM for 24 h	H9c2	• ↑ cell viability dose-dependently • ↓ IL-1β, IL-18 & TNF-α with increase in concentration • ↓ TLR4, MyD88, p-IKKα/ IKKα, p-IKKβ/ IKKβ, p-Iκ Bα/ Iκ Bα, p-NFκB/ NFκB expression dose-dependently • ↓ NLRP3, ASC, caspase-1 expression with ↑ dose	[77]
	8 µM for 12 h	HL-1	• ↑ cell viability • ↓ cellular apoptosis	[78]
	0.1–100 µM for 2 h	H9c2	• Inhibited H_2_O_2_-induced cell death • 15–20 µM ↓ LDH release and restored cell morphology • Reverse H_2_O_2_-induced peroxiredoxin II expressions	[79]
**Mangiferin**	1, 2, 4, 8, & 16 µM	H9c2	• Inhibited oxidative stress • ↓ TNF-α, IL-6, IL-1β in cell supernatant	[36]
**Morin**	12.5, 25, & 50 µM for 12 h before H/R (720 − 60)	H9c2	• ↑ Cell viability after H/R and ↓ LDH release in medium • ↓ cellular apoptosis i.e. ↑ Bcl-2, ↓ Bax & Bax/Bcl-2 ratio via mRNA expression and apoptosis rate • ↓ Cytochrome c, APAF-1, cleaved caspase 9/ 3 protein	[62]
**Naringenin**	40, 80 & 160 µM; 24 h before H/R	H9c2	• ↑ Cell viability, ↑ Bcl-2 (anti-apoptotic protein) • ↓ caspase-3 & Bax (pro-apoptotic proteins) • Reversed ER stress: upregulate Glucose-regulated 78, C/EBP homologous & cleaved caspase-12 proteins. • ↑ cleavage activating transcription factor 6 (ATF6) • ↑ p-ERK and IRE1a.	[44]
	40, 80, &160 µmol/L for 6 h before H/R	H9c2	• ↑ Cell viability • ↓ Apoptosis (↓ Caspase-3 & ↓ Cleaved caspase-3)	[45]
**Nobiletin**	12.5, 25, and 50 µM for 24 h before H/R (180–360)	H9c2	• ↑ cell viability and ↓ apoptosis • ↓ ROS, MDA, TNF, IL-1β, IL-6 • Activate Akt/ GSK3β pathways (↑expression)	[93]
	µM 2 h before OGD surgery	H9c2	• Maintains cell viability and ↓ cellular apoptosis	
**Quercetin**	40 µM for 24 h (360 − 30)	H9c2	• ↓ iNOS expression and ↓ DHE intensity (less ROS) • ↓ Apoptotic cells and cleaved-caspase 3 expression • ↓ % of NFκBp65 and p-IκBα expression	[91]

*Note* the hypoxia reoxygenation (H/R) timing is mentioned in minutes (mins) (e.g. 60 min of hypoxia followed by 180 min of reoxygenation in in vitro)

## Conclusion

Multiple preclinical studies have demonstrated and provided evidence for cardio-protective applications of flavonoids in attenuating myocardial IR injury, and also shown their role in pleiotropic pathways such as the inherent ability to ameliorate oxidative stress, inhibit apoptosis, and reduce inflammation. The antioxidant activity is influenced by increasing levels of glutathione and by decreasing levels of superoxide dismutase and malondialdehyde. Moreover, the anti-inflammatory role of flavonoids is governed by downregulating transcription of NF-κB subsequently inhibiting the generation of various pro-inflammatory cytokines (IL-6, IL-1β, and TNF-α). In addition, the anti-apoptotic activity of flavonoids is accomplished by inhibiting cytosolic proteases including caspase-3, caspase-8, and caspase-9.

Even though numerous preclinical studies have evidenced the potent characteristics of flavonoids in the amelioration of IR injury, a comprehensive assessment of their dosages and potential adverse effects is essential before any recommended therapeutic utilization. Furthermore, given the pivotal role that flavonoids play, there is a pressing imperative to explore novel reservoirs of these bioactive compounds. Diverse botanical specimens historically utilized in Ayurveda, Siddha, and Unani medicinal traditions are replete with flavonoids, thus warranting deliberate investigation for their extraction. Thus, there is a dire need for clinical studies for the extensive exploration of flavonoids for their potential role in myocardium protection.

## Electronic supplementary material

Below is the link to the electronic supplementary material.

Supplementary Material 1

## Data Availability

The data of this study was collected from online resources only.

## Declarations

### Declarations of generative AI

No AI tool was used to generate data and preparation of the manuscript (including text, tables, figures etc.).

## Abbreviations

8-OHdG: 8-hydroxy-2’ -deoxyguanosine
APAF: Apoptotic protease activating factor
ASC: Apoptosis-associated speck-like protein containing CARD
AST: Aspartate transaminase
BAX: Bcl-2–associated X protein
Bcl-2: B-cell lymphoma 2
CK-MB: Creatine Kinase-Myocardial Band
CPK: Creatine phosphokinase
CREB: Cyclic-AMP response-binding protein
cTnI: Cardiac troponin I
DHE: Dihydroethidium
ERK: Extracellular signal-regulated kinase
GPx: Glutathione peroxidase
GR: Glutathione reductase
GSH: Reduced glutathione
GSK: Glycogen synthase kinase
HR: Heart Rate
IHD: Ischemic Heart Disease
IKKα: Inhibitory Kappa B Kinaseα
IL: Interleukin
IR: Ischemia Reperfusion
IRE1α: Inositol-requiring transmembrane kinase/endoribonuclease 1α
LDH: Lactate Dehydrogenase
LV dp/dt: Rate of change in left ventricular pressure
LVEDP: Left ventricular end diastolic pressure
LVF: Left Ventricular Function
MDA: Malonaldehyde
MIAT: Myocardial infarction associated transcript
MIRI: Myocardial Ischemia-Reperfusion Injury
MPO: Myeloperoxidase
NLRP3: NLR family pyrin domain containing 3
NO: Nitric oxide
Pre-op: Pre operation / before surgery
RAGE: Receptor of advanced glycation end-products
ROS: Reactive Oxygen Species
SOD: Super Oxide Dismutase
TBRAS: Thiobarbituric acid reactive substance
TLR: Toll-like receptor
TNF: Tumour Necrosis Factor
