# Supplementary material for: Flavonoids as therapeutics for myocardial ischemia-reperfusion injury: a comprehensive review on preclinical studies
Source: Lab Anim Res. 2024 Sep 5;40:32. doi: 10.1186/s42826-024-00218-2 (PMC11376054; doi:10.1186/s42826-024-00218-2)
Supplement: Supplementary file 1 — Supplementary Material 1 [file 42826_2024_218_MOESM1_ESM.docx]

To 7^th^ August, 2024

The Editor-in-Chief

Laboratory Animal Research

Subject: Submission of Revised Review Article for Publication as per the reviewers

Dear Sir/ Madam,

Thank You for providing me the opportunity to proof-read my manuscript titled “**Flavonoids for the Treatment of Myocardial Ischemia-Reperfusion Injury: A Systematic Analysis of Preclinical Studies**” and suggesting the changes, to shape the manuscript as per the journal guidelines. I am hereby submitting the revised version of this Manuscript (Review Article), following your suggestions for further evaluation and processing. If you have any other suggestions for the improvement of the manuscript please contact me. I am happy to do the revisions for improvement of this review article of scientific need.

Thank you again for accepting this article and valuable suggestions for the improvement of this manuscript.

Thanking you

With Regards and Best Wishes

**Dr. D. S. Arya**

Professor and Head

Department of Pharmacology

All India Institute of Medical Sciences

New Delhi-110 029, India

Email: [dsarya16@aiims.edu](mailto:dsarya16@aiims.edu)
